# Combined effects of EMP and RF field on emotional behavior in mice

**DOI:** 10.3389/fpubh.2023.1087161

**Published:** 2023-03-16

**Authors:** Tongzhou Qin, Liyuan Liu, Xing Wang, Ling Guo, Jiajin Lin, Junze Du, Yizhe Xue, Panpan Lai, Yuntao Jing, Guirong Ding

**Affiliations:** ^1^Department of Radiation Protection Medicine, School of Preventive Medicine, Air Force Medical University, Xi'an, China; ^2^Ministry of Education Key Lab of Hazard Assessment and Control in Special Operational Environment, Xi'an, China

**Keywords:** RF field, EMP, anxiety-like behavior, autophagy, amygdala

## Abstract

**Background:**

Recently, concerns about the combined effects of electromagnetic field (EMF) in daily living and occupational environment are rapidly growing.

**Methods:**

In this study, we investigated the combined effects of 1-week exposure to electromagnetic pulse (EMP) at 650 kV/m for 1,000 pulses and 4.9 GHz radiofrequency (RF) at 50 W/m^2^ for 1 h/d in male mice. Open field test, tail suspension test and Y-maze were applied to evaluate anxiety, depression-like behaviors and spatial memory ability, respectively.

**Results:**

It was found that compared with Sham group, combined exposure to EMP and RF induced anxiety-like behavior, increased the level of serum S100B and decreased the level of serum 5-HT. The results of quantitative proteomic and KEGG analysis showed that the differentially expressed proteins in hippocampus were enriched in Glutamatergic and GABAergic synapse after combined exposure group, which were verified by western blot. In addition, an obvious histological alteration and autophagy-associated cell death were observed in amygdala instead of hippocampus after combined exposure to EMP and 4.9 GHz RF.

**Conclusion:**

Combined exposure to EMP and 4.9 GHz RF could induce emotional behavior alteration, which might be associated with Glutamatergic and GABAergic synapse system of hippocampus and autophagy in amygdala.

## 1. Introduction

The emerging 5G (the 5th Generation) mobile network provides faster transmission speed and increasingly massive mobile data usage, which brings much convenience to our life. Radiofrequency electromagnetic radiation (RF-EMR) is widely used in the living environment with the frequencies ranging from 3 kHz to 300 GHz. 5G network operates across the RF spectrum, which can be classified as “low band” (below 1 GHz); “middle band” (1–6 GHz range); “high band” (~30–300 GHz in the millimeter wave portion) (1), the 4.9 GHz is “middle band” of 5G network in China and has been used in many cases. At the same time, numerous people are inevitably being exposed to RF field during mobile phone usage. Therefore, much attention has been paid to the potential side effects of RF field on human health. In 2011, the International Agency for Research on Cancer (IARC) announced RF to be the “possible carcinogenic to humans”, group 2B (2). Evidence from a large number of literature have revealed the health-related harmful effects (3–6) caused by RF, moreover, the brain is a sensitive target organ to RF exposure and the adverse effect of which is more severe as compared to other organ systems (7, 8).

Electromagnetic pulse (EMP) is a short high-voltage pulse which characterized by spectral bandwidth ranging from extremely low frequency up to 1.5 GHz with extremely fast rise time (9). It was found that EMP exposure could increase the permeability of blood brain barrier, and enhance the drug delivery to brain (10–13). In addition, it had therapeutic effects on oncology in clinical trials (14, 15). Besides, Terahertz (one of electromagnetic pulsed spectrum) has been trials application in cancer detection and biological tissue discrimination (16). Therefore, EMP is a promising method for medical detection and treatment. Due to the special properties, there are concerns that EMP exposure may have potential harmful effects on human health, especially for individuals who work with or may be exposed to electronic components in occupational environment. Till now, the biological effects of EMP have not been fully understood. Jiang et al. found that EMP exposure could cause the increase of beta amyloid protein (Aβ) and beta site App cleaving enzyme (BACE1) in hippocampus, which led to Alzheimer's disease-like (AD-like) behavior in rats (17, 18). These findings indicated that EMP exposure may have potential clinical application and it may exert potential hazard on brain functions.

To our knowledge, most studies have focused on the effects of single electromagnetic radiation, only a few studies have investigated the combined effects from multi-frequency or multi-variations radiation (19). 4.9 GHz RF is one of commonly used signals for 5G network communication in China, we previously found that long-term single exposure (consecutively 21 days) to 4.9 GHz RF could induce emotional behavior alteration in mice (20). Till now, the combined effects of EMP and 4.9 GHz RF have not been reported. Therefore, in the present study, we aimed to investigate the combined effects of 4.9 GHz RF and EMP on emotional behaviors and spatial memory ability in adult mice.

## 2. Materials and methods

### 2.1. Animals

Healthy adult male C57BL/6 mice (8 weeks, 18.7 ± 2.4) g, purchased from the Laboratory Animal Center of Air Force Medical University (Xi'an, China), were randomly divided into Sham group (Sham), 4.9 GHz RF exposure group (4.9 GHz RF), Electromagnetic pulse exposure group (EMP) and combined exposure group (EMP + 4.9 GHz RF) (*n* = 12 for each group). Before the exposure, all mice were housed under controlled conditions (temperature 23 ± 2°C, humidity 50 ± 2%, 12-h light and 12-h dark cycle) and had access to water and food. The animals were allowed to adapt to the environment for 1 week before the experiment, and the body weight of mice was measured every 2 days. All the procedures in this study were conducted in accordance with the ethical guidelines of Animal Welfare Committee of Air Force Medical University (IACUC-20210105).

### 2.2. Exposure to RF and/or EMP

**Figure 1:**
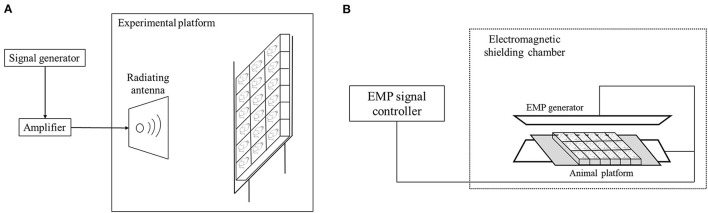
Schematic illustration of exposure system setting. **(A)** RF exposure system set-up. **(B)** EMP exposure set-up.

The 4.9 GHz RF exposure system is mainly comprised of a signal generator, an amplifier and a radiating antenna. The EMP generator (Northwest Institute of Nuclear Technology) was placed in an electromagnetic shielding chamber, and the animals were placed in a platform between the generator. The EMP pulses were generated by a spark gap generator and transmitted to the animal platform in the shielding chamber (Figure 1). During the exposure, the mice were individually placed in a transparent plexiglass box (45 cm × 8 cm × 7 cm) with small ventilation holes on every side of the walls to make sure the mice breath well. For 4.9 GHz RF exposure, the average Power Density (PD) was 50 ± 2.5 W/m^2^, which was measured by an electromagnetic field meter (PMM8053A, PMM Costruzioni Electtroniche Centro Misure Radio Electriche S.r.l., Milan, Italy), the distance between animals and RF antenna was 0.7 m. For EMP exposure, the electric field intensity was 650 kV/m with 1,000 pulses in total, the pulse repetition rate was 2 pulses per second (the time interval was 0.5 s) and the total exposure time was about 10 min/d. For combination exposure group, the mice were exposed to 4.9 GHz RF for 1 h/d and EMP for 10 min/d. The rectal temperature of mice was measured before and after exposure of EMP and RF exposure, and the temperature rise was below 1°C. The mice in the Sham group were also placed in the similar plexiglass box and treated the same way as those in exposure groups, while the power system of RF and EMP generator were switched off.

### 2.3. Open field test

Open field test (OFT) is commonly used to evaluate animals' locomotor activity, exploratory and anxiety-like behavior in novel environment. The OFT was conducted as described previously (21). Briefly, the mice (*n* = 12 for each group) were separately placed in the center of open field (50 cm × 50 cm × 40 cm), and were allowed to move freely. The activity of mice in the open field was recorded by a computer-operated tracking system during a 5-min session (Ethovision XT 15.0 Noldus software, Holland). After each session, all feces and urine were removed, 75% alcohol solution was used to clean the apparatus to prevent the odor traces. The accumulative locomotion distance, total time spent in the central areas and number of central area entries were analyzed.

### 2.4. Y-maze test

The Y-maze was applied to evaluate animals' responsiveness to novel environment and spatial memory. It was performed based on previous study (22) with proper modifications. Briefly, at the first phase, one of the arms was blocked, each mouse (*n* = 12 for each group) was gently placed into one of the open arms, and was allowed to explore the maze for 10 min without disturbance. One hour later, all arms were opened for exploration, each mouse was placed into the same arm as the first phase, and let it explore for 5 min. After each trial, the feces and urine were cleaned and the walls of maze were wiped with 75% alcohol solution. During the test, the activity of mice was recorded with digital video tracking system (Ethovision XT 15.0 Noldus software, Holland). Percentage of time spent in the novel arm and number of novel arm entries were analyzed.

### 2.5. Tail suspension test

The procedure of tail suspension test (TST) was based on previous study (23). Briefly, mice (*n* = 12 for each group) were suspended with adhesive tape on the tail, and the distance between mouse's nose and apparatus floor was about 25 cm. In order to prevent the mice from climbing to their tail during the experiment, a small plastic cylinder (3 × 0.3 cm) was placed on the tail. Each mouse was suspended for 6 min, and the immobility time was recorded during the last 4 min. The movement of each mouse during the whole test was recorded with video tracking system (Ethovision XT 15.0 Noldus software, Holland). All data were analyzed by the observer who was blind to the group assignment.

### 2.6. Hematein-Eosin and Nissl stain

After the exposure procedure, all mice were deeply anesthetized with 1% sodium pentobarbital (60 mg/kg) and perfused with phosphate buffered saline (PBS, PH7.4). The whole brain was isolated and fixed in 4% paraformaldehyde for 1 day. The brains (*n* = 3 for each group) were sliced with the thickness of 4 μm by Leica RM2135 rotary microtome (Leica, Germany). Subsequently, the sections were stained with Hematein-Eosin (HE) and Nissl according to routine procedure. The morphology of the brain was observed by Leica DMI4000B microscope (Leica, Germany).

### 2.7. Tandem mass tags quantitative proteomic analysis

The protein expression profile of hippocampus (*n* = 3 for each group) after exposure to EMP and 4.9 GHz RF were assessed by high performance liquid chromatography (HPLC) with tandem mass tags (TMT) proteomic analysis, which were carried out by PTM-BIO Co. Ltd. (Hangzhou, China). Briefly, the samples were lysed by ultrasound and centrifuged to remove cell debris, and the protein concentration was determined using BCA assay. The same amount of sample protein was taken for enzymatic hydrolysis, then the protein digestion and labeling with TMT reagent were performed according to the operating instructions. The peptides were fractionated by high pH reverse-phase HPLC on an Agilent 300Extend C18 chromatographic column, then the peptides were separated by an ultra-high performance liquid system and ionized after being injected into NSI ion source, and analyzed by Orbitrap Exploris™ 480 mass spectrometry.

### 2.8. Bioinformatics analysis

Gene Ontology (GO) annotation proteome and Kyoto Encyclopedia of Genes and Genomes (KEGG) analysis were derived from the Uniprot-GOA database (http://www.ebi.ac.uk/GOA/) and (http://www.genome.jp/kegg) database, respectively. Briefly, converting identified protein ID to UniProt ID and then mapping to GO database, the InterProScan software would be used to annotated protein's GO function if identified proteins were not annotated by UniProt-GOA database. Then the proteins were classified by GO annotation based on three categories: biological process, cellular component and molecular function. For KEGG pathways analysis, KEGG online service tools KAAS was used to annotated protein's KEGG database description, then mapping the annotation result on KEGG pathway database. For each category, a two-tailed Fisher's exact test was employed to test the enrichment of the differentially expressed protein (DEPs) against all identified proteins, the DEPs were screened and obtained using the cut-off of fold change >1.2 and *P* < 0.05. The GO with a corrected *P-*value < 0.05 is considered significant.

### 2.9. Enzyme linked immunosorbent assay (ELISA)

Blood samples were taken from the left ventricle of the heart. After 2 h, centrifuge at 3,000 rpm for 15 min at 4°C and collect serum. The expression level of S100 calcium-binding protein B (S100B) and Serotonin (5-HT) were measured with ELISA kit (Sinoukbio, Beijing, China; *n* = 10 for each group) according to the manual.

### 2.10. TUNEL staining assay

The hippocampal cells apoptosis level was assessed by terminal deoxynucleotidyl transferase (TdT) enzymaticated dUTP nick end labeling (TUNEL) assay using Cell Death Detection Kit (Servicebio, Wuhan, China) according to the manual. Briefly, after routine deparaffinization and antigen recovery, the sections were permeabilized with Triton X-100 (Beyotime, China), followed by 30 μl TUNEL reaction mixture for 60 min at 37°C. 3 fields were randomly chosen in each group for analysis using a fluorescence microscope (Leica), and the apoptosis rate was calculated with Image J software.

### 2.11. Immunofluorescence staining

The brain tissues (*n* = 3 for each group) were carefully dissected out from the mice fixed in 4% PFA for 24 h, and then dehydrated in 30% sucrose solution for at least 48 h. Hippocampal coronal slices were sliced at 20 μm thickness and stored at −20°C before staining. Sections of brain tissue were treated with 1% Bovine serum albumin (BSA) for 1 h, then the sections were incubated with LC3 (1:200, rabbit polyclonal antibody, Proteintech, USA) at 4°C overnight. After washing 3 times with PBS, the sections were incubated in a dark place with fluorescent secondary antibodies: Alexa Fluor 594-labeled goat anti-rabbit IgG (1:200) for 1 h at room temperature. All sections were later counterstained with DAPI to label nuclei at room temperature for 8 min. Finally, an anti-fluorescence quenching agent (24) was used to seal the slides. Digital images were captured with a Leica DMI4000B microscope (Leica, Germany).

### 2.12. Western blotting

The total proteins of hippocampus tissues (*n* = 3 or 4 for each group) were obtained with the whole protein extraction kit (Cat KGP250, KeyGen Biotech, Nanjing, China), and the concentration of hippocampal total proteins was measured by bicinchoninic acid (BCA) protein assay kit (Beyotime, China). The protein samples were separated by 10–12% sodium dodecyl sulfate-polyacrylamide gel electrophoresis (SDS-PAGE) and transferred onto polyvinylidene fluoride (PVDF) membrane. The membranes were blocked with 5% non-fat milk for 2 h at room temperature and incubated overnight at 4°C with the following primary antibodies: rabbit mAb anti-β-actin (#13E5; Cell Signaling Technology), rabbit pAb anti-β-tubulin (#10094-1-AP; Proteintech), rabbit pAb anti-Bax (#50599-2-Ig; Proteintech), rabbit pAb anti-Bcl-2 (#12789-1-AP; Proteintech), rabbit pAb anti-Cleaved caspase 3 (#AF7022; Affinity), rabbit pAb anti-LC3-II (#14600-1-AP; Proteintech), rabbit mAb anti-Glutamate Receptor 1 (ab183797; Abcam), rabbit mAb anti-Gamma aminobutyric acid (GABA) BR1 (ab238130; Abcam), rabbit mAb anti-GABA BR2 (ab75838; Abcam). After rinsing with TBS-0.1% Tween 20, the membranes were then incubated with HRP-conjugated goat anti-rabbit (1:5,000, CWBIO, China) for 2 h at room temperature. Finally, protein bands were detected using enhanced chemiluminescent kit (Beyotime, China). Gray value analysis was performed using Quantity One 4.6.2 software (Bio-Rad, Milan, Italy).

### 2.13. Statistical analysis

All data were presented as mean ± standard error of mean (SEM) and all graphs were generated using GraphPad Prism 8.0 software (San Diego, CA, United States). Normality of data was tested by Shapiro-Wilk test first, for normally distributed data, a two-tailed Student's *t*-test or one-way analysis of variance (ANOVA) followed by Tukey test for multiple comparisons was used. For non-normal distribution data, non-parametric analysis was used. The data analysis was performed by the individual blinded to the experiment. *P* < 0.05 was considered statistically significant.

## 3. Results

### 3.1. The combined effects of exposure to EMP and 4.9 GHz RF on the spatial learning and emotionality of mice

**Figure 2:**
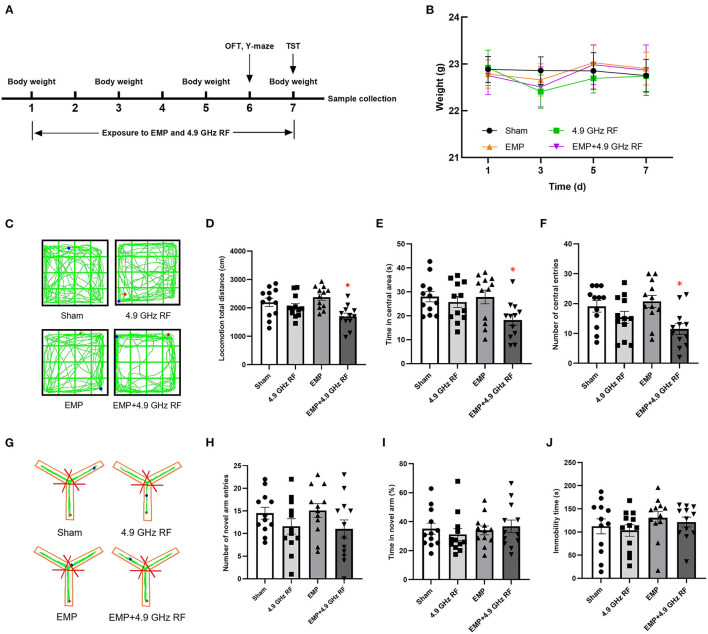
Effects of combined exposure to EMP and 4.9 GHz RF on general health and ethology. **(A)** Time schedule of exposure for mice. **(B)** Body weight of mice. *n* = 12 for each group. **(C)** The representative original trace of OFT. **(D)** Locomotion total distance in open field. **(E)** Time spent in central area. **(F)** Number of central entries. **(G)** The representative original trace of Y-maze. **(H)** Number of novel arm entries. **(I)** Percentage of time spent in novel arm. **(J)** Immobility time. *P < 0.05 vs. Sham group. *n* = 12 for each group.

Figure 2A shows the time schedule of EMP and 4.9 GHz RF exposure for mice. All the mice in each group were in good body conditions. The mice in the exposure groups showed a slight decrease in body weight on Day 3, after that, the weight gradually returned to the normal level (Figure 2B).

The results of OFT showed that the locomotion total distance in the open field, the time spent in central area and number of central area entries significantly decreased in combined exposure group, compared with Sham group (Figures 2C–F). No significant differences were found between the Sham group and single exposure groups. The above results indicated that combined exposure of EMP and 4.9 GHz RF under current conditions could induce anxiety-like behavior and reduce locomotor activity of mice.

The ability of spatial learning and memory of mice was assessed by Y-maze. The results showed that there were no significant differences in the percentage of time spent in novel arm and number of novel arm entries (Figures 2G–I). These results indicated that under current conditions, single or combined exposure to EMP and 4.9 GHz RF had no obvious effects on the spatial learning and memory ability of mice.

The result of TST (Figure 2J) showed that compared with Sham group, the exposure groups had no significant difference in the immobility time, which indicated that single or combined exposure to EMP and 4.9 GHz RF for 1-week would not induce the depression-like behavior of mice.

### 3.2. Differentially expressed proteins and gene oncology analysis

**Figure 3:**
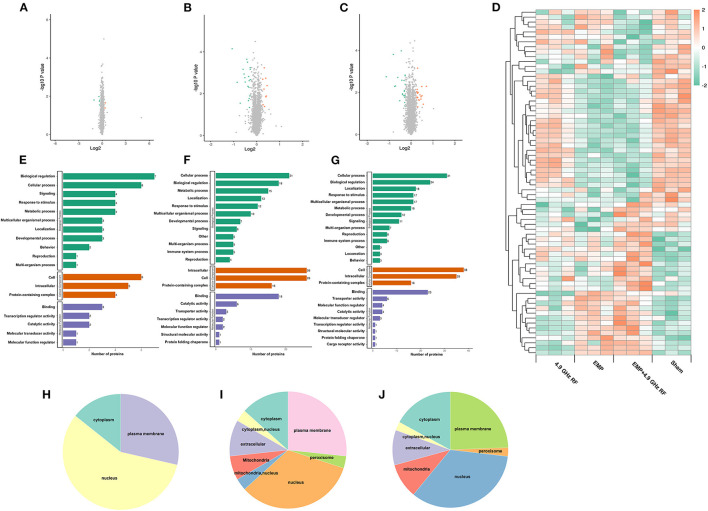
Bioinformatics analysis of differentially expressed proteins. The volcano plot of DEPs in 4.9 GHz RF exposure group, EMP exposure group and combined exposure group **(A–C)**. Red points represent significantly up-regulated proteins and green points represent significantly down-regulated proteins. **(D)** Hierarchical clustering of DEPs in exposure groups and Sham group. GO enrichment analysis of DEPs in **(E)** 4.9 GHz RF exposure group; **(F)** EMP exposure group and **(G)** combined exposure group. **(H)** Subcellular localization prediction of 4.9 GHz RF exposure group; **(I)** EMP exposure group and **(J)** combined exposure group.

To explore the underlying genetic mechanisms involved in behavioral changes, we performed TMT-based quantitative proteomics analysis using hippocampal samples from each group. A total of 5,293 quantitative proteins and 33,185 unique peptides were identified. There were 78 genes that met the criteria of fold change >1.2 and *P* < 0.05 in *t*-test, of which 30 were significantly up-regulated and 48 were significantly down-regulated. The DEPs of up- and down-regulated are presented as volcano plots in Figures 3A–C. The hierarchical clustering of DEPs was shown as a heat map in Figure 3D.

Gene oncology (GO) enrichment analysis of DEPs showed that all DEPs were classified into three categories (Figures 3E–G): Biological Process (BP), Cellular Component (CC) and Molecular Function (MF). The DEPs were mainly enriched in cellular process and biological regulation in BP; CC such as intracellular and cell; DEPs were mainly enriched in binding process in MF. We also found that the majority of DEPs were localized to nucleus, followed by plasma membrane and cytoplasm (Figures 3H–J).

### 3.3. KEGG enrichment analysis of differentially expressed proteins

**Figure 4:**
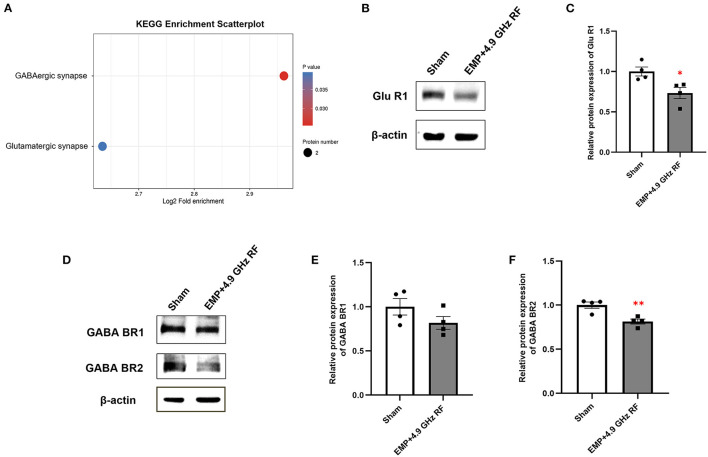
KEGG analysis of DEGs in the hippocampus of mice. **(A)** KEGG enrichment analysis of DEPs. **(B)** Western blot analysis of Glu R1. **(C)** Relative protein expression level of Glu R1. Western blot analysis of GABA BR1 and GABA BR2 **(D)** and relative protein expression levels **(E, F)**. *n* = 4 for each group, *P < 0.05, **P < 0.01 vs. Sham group.

Kyoto Encyclopedia of Gsenes and Genomes (KEGG) enrichment analysis of DEPs in hippocampus was shown in Figure 4A. There were only two down-regulated pathways enriched in combined exposure groups compared with Sham group, including Glutamatergic synapse and GABAergic synapse. Importantly, GABAergic system is essential for modulating the plasticity of neural network through GABA receptors, and glutamate is the main excitatory neurotransmitter in central nervous system (CNS). We speculated that GABAergic and Glutamatergic synapse system might be involved in the effects of EMP and 4.9 GHz RF combined exposure to adult mice. We then verified the relative protein expression of representative Glutamatergic and GABAergic receptors by western blot (Figures 4B–F). The results showed that in combined exposure group, the protein expression level of Glu R1 was significantly down-regulated compared with Sham group. The relative protein expression of GABA BR2 was decreased significantly, while the level of GABA BR1 remained unaltered. The results were consistent with KEGG enrichment of DEPs, indicating that EMP and 4.9 GHz RF combined exposure could affect GABAergic and Glutamatergic synapse system in CNS, which might be involved in behavioral changes in mice.

### 3.4. The combined effects of exposure to EMP and 4.9 GHz RF on S100B and 5-HT in mice serum

**Figure 5:**
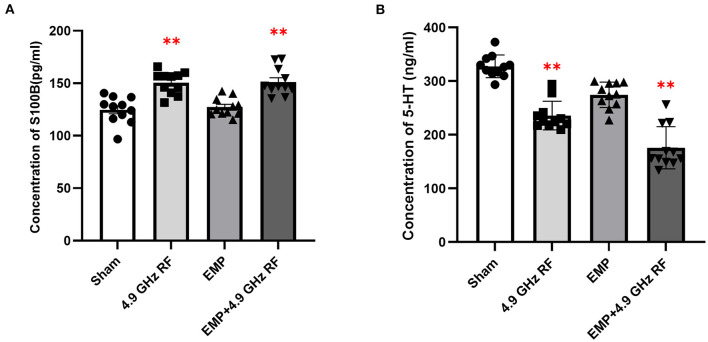
Effects of combined exposure to EMP and 4.9 GHz RF on the levels of S100B and 5-HT in serum determined by ELISA. **(A)** The concentration of S100B in serum. **(B)** The concentration of 5-HT in serum. *n* = 11 for each group. **P < 0.01 vs. Sham group.

The results of ELISA showed that compared with Sham group (Figure 5A), 4.9 GHz RF single exposure and combined exposure significantly increased the level of S100B in mice serum, while it did not change obviously in EMP single exposure group. The level of 5-HT significantly reduced after combined exposure to 4.9 GHz RF and EMP, and it also obviously decreased in RF single exposure group (Figure 5B).

### 3.5. The combined effects of exposure to EMP and 4.9 GHz RF on the histology of the brain

**Figure 6:**
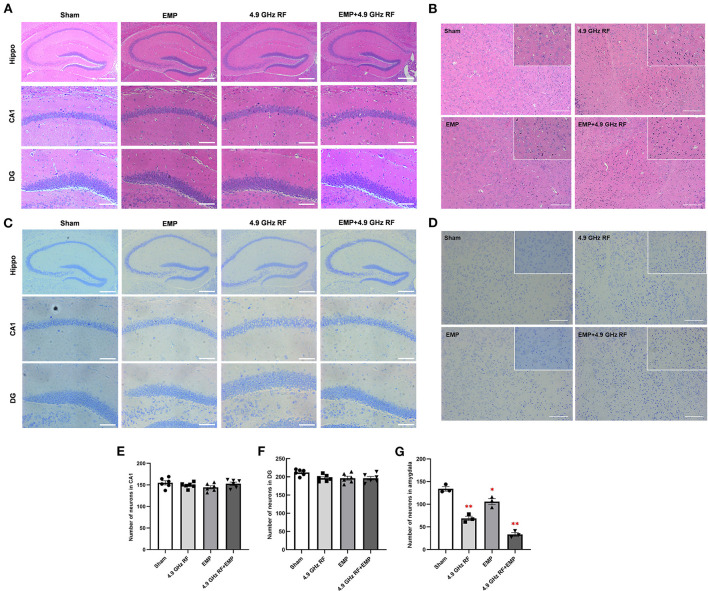
The combined effects of exposure to EMP and 4.9 GHz RF on the histology of brain. **(A)** HE staining of hippocampal morphology in CA1 and DG regions. **(B)** HE staining of amygdala. **(C)** Nissl staining of hippocampal CA1 and DG regions. **(D)** Nissl staining of amygdala. The number of neurons in CA1 region **(E)** and DG region **(F)**, six random fields were chosen for each group. **(G)** The number of neurons in amygdala. *n* = 3 for each group. Scale bar = 200 μm for hippocampus (Hippo); scale bar = 100 μm for amygdala; scale bar = 50 μm for CA1, DG and magnified images of amygdala. *P < 0.05, **P < 0.01 vs. Sham group.

We then observed the histology of hippocampus and amygdala in the brain. The results of HE and Nissl staining (Figures 6A, C) showed that there were no obvious histological differences in hippocampal CA1 and DG after the exposure to EMP and 4.9 GHz RF, besides, the number of neurons in hippocampal CA1 and DG regions was not altered (Figures 6E, F). However, numerous deep staining cells, karyopyknosis and necrosis were found in amygdala of 4.9 GHz RF single exposure and combined exposure groups (Figures 6B, D), and the number of neurons in amygdala significantly decreased (Figure 6G). The above results suggested that single or combined exposure to EMP and 4.9 GHz RF had no obvious effects on the morphology of hippocampus while it could induce structural damage and neuronal loss in amygdala.

### 3.6. The combined effects of exposure to EMP and 4.9 GHz RF on apoptosis of the brain

**Figure 7:**
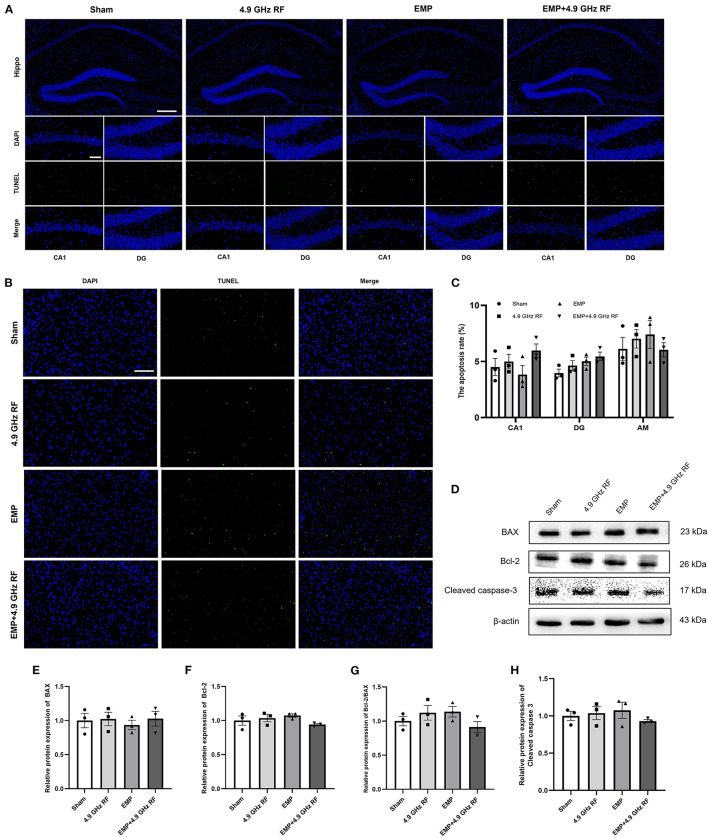
The combined effects of exposure to EMP and 4.9 GHz RF on apoptosis in hippocampus and amygdala. **(A)** Result of TUNEL staining of hippocampus. **(B)** Result of TUNEL staining of amygdala. Scale bar = 100 μm for Hippo, scale bar = 50 μm for CA1, DG and amygdala regions. **(C)** Quantitative analysis of apoptosis rate. **(D)** The levels of apoptosis-related proteins were detected by western blot. Relative protein expression levels of Bax **(E)**, Bcl-2 **(F)**, Bcl-2/Bax **(G)** and Cleaved caspase 3 **(H)** in hippocampus. *n* = 3 for each group.

We then conducted TUNEL and Western blot to determine whether EMP and 4.9 GHz RF could induce cells apoptosis in the brain. The results of TUNEL staining revealed that the apoptosis rate of hippocampal cells in CA1 and DG regions and amygdala had no significant differences compared with Sham group (Figures 7A–C). The apoptosis-related proteins were further verified by Western blot. The results suggested that, single or combined exposure of EMP and 4.9 GHz RF did not affect the expression of Bax, Bcl-2 and Cleaved caspase 3 in hippocampus (Figures 7D–H). The above results indicated that EMP and 4.9 GHz RF exposure had no obvious effect on the cells apoptosis in hippocampus and amygdala.

### 3.7. The combined effects of exposure to EMP and 4.9 GHz RF on autophagy of the brain

**Figure 8:**
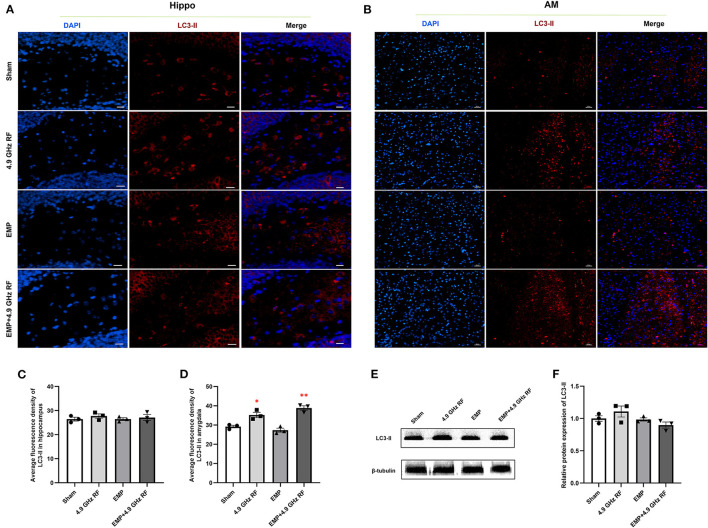
The combined effects exposure to EMP and 4.9 GHz RF on autophagy in hippocampus and amygdala. Immunofluorescence staining of LC3-II in hippocampus **(A)** and amygdala **(B)**. Scale bar = 100 μm for AM, scale bar = 50 μm for Hippo. Average fluorescence density of LC3-II in hippocampus **(C)** and amygdala **(D)**. **(E, F)** Western blot analysis and relative protein expression of LC3-II in hippocampus. *n* = 3 for each group, *P < 0.05, **P < 0.01 vs. Sham group.

The results of immunofluorescence showed that EMP and 4.9 GHz RF single or combined exposure did not induce autophagy in hippocampus, and the average fluorescence density of marker for autophagy LC3-II in hippocampus had no significant differences compared with Sham group (Figures 8A, C). The level of autophagy in amygdala in 4.9 GHz RF single exposure was increased compared with Sham group, the similar result was also observed in EMP and 4.9 GHz RF combined exposure group (Figure 8B), which was evidenced by the significantly increased average fluorescence density of LC3-II in amygdala, compared with Sham group (Figure 8D). We then further investigated the protein expression level of LC3-II in hippocampus by western blot, no significant difference was found between the exposure groups and Sham group, which was consistent with the result of immunofluorescence (Figures 8E, F). These results indicated that single or combined exposure to EMP and 4.9 GHz RF could induce cell autophagy in amygdala but not hippocampus.

## 4. Discussion

In this study, we investigated the effects of combined exposure to EMP and 4.9 GHz RF for 7 days on the spatial memory and emotional behavior of mice. The results of OFT showed that the anxiety-like behavior was not induced after single exposure to EMP or 4.9 GHz RF. However, after combined exposure to EMP and 4.9 GHz RF, the locomotion distance, cumulative time and central area entries significantly decreased, compared with Sham group, which indicated that combined exposure could induce anxiety-like behavior. Júnior et al. (25) found that 3-day continuous exposure to 1.8 GHz RF did not induce anxiety-like behaviors in rats. Besides, Barthelemy et al. (26) found that 900 MHz RF (whole-body SAR were 0, 1.5 and 6 W/kg) exposure could not induce the anxiety-like behavior in rats. Nevertheless, some scholars found inconsistent results concerning the effects of RF single exposure on anxiety-like behavior (27, 28). The contradicting results may be attributed to differences in frequencies, exposure method and duration. Till now, no literatures were available about the effects of EMP exposure on anxiety-like behaviors of animals. We then investigated the depression-like behavior of mice by TST, the result showed that single or combined exposure to EMP and 4.9 GHz RF could not induce depression-like behaviors of mice. Consistent with our finding, Zhang's study revealed that exposure to 1.8 GHz RF for 4-week did not affect the depression-like behavior of adult mice (29). Regarding the effects of RF exposure on depression-like behavior, the literature is relatively rare, which needs further exploration.

Y-maze is a hippocampal-dependent test which is widely used to evaluate spatial memory in mice. Our study found that single and combined exposure to EMP or 4.9 GHz RF had no obvious effects on spatial memory of mice, which was consistent with Keleş and Sienkiewicz's studies (30, 31). However, inconsistent findings were also reported. Zhu et al. previously revealed that combined exposure to 1.5 and 4.3 GHz microwave could induce decline of learning and memory ability in rats (32). In Nittby et al.'s study (33), it was found that the memory functions reduced after exposure to 900 MHz RF (whole-body SAR was 0.6 and 60 mW/kg) for 2 h/week for 55 weeks. Interestingly, it was reported that exposure to 900 MHz mobile phone signal (whole-body SAR 0.3 or 3.0 W/kg) for 5 weeks, 2 h/d, 5 days/week, could significantly improve learning and memory ability (34). The reports about the effects of EMP on animals' learning and memory are rare. One study found that EMP exposure (field strength 50 kV/m, repetition rate 100 Hz) could cause long-term cognition and memory impairment in rats (18). Another study revealed that the learning ability of mice significantly decreased after EMP exposure (peak-intensity 400 kV/m, rise-time 10 ns, pulse width 350 ns), and it recovered at 2 d after exposure (35).

In this study, KEGG analysis revealed that several differentially expressed proteins were significantly down-regulated in the processes of Glutamatergic and GABAergic synapse, which indicated that combined exposure could induce an inhibitory effect on GABAergic and Glutamatergic system. Glutamate is the main excitatory neurotransmitter in CNS, some neuronal functions mainly rely on Glutamatergic synapses, including synaptic transmission, neuronal migration, excitability and so on (36). GABA is the most abundant inhibitory neurotransmitter, and GABAergic system has pivotal role in modulating activity and plasticity of neural networks during development (37). Moreover, it has been reported that GABA-mediated neurotransmission has a crucial role in anxiety, but the data are limited (38). Mombereau et al. found that GABA B1 receptor deficient mice performed more anxiety-like behavior than wild-type mice (39). Another study revealed that Glutamate represents a key molecule associated with comorbidities between neurological disorders (40). Therefore, Glutamatergic and GABAergic synapses were considered to be involved in synapse plasticity and help facilitate brain remodeling (41). Based on the present findings, we assume that EMP and 4.9 GHz RF combined exposure can affect Glutamatergic and GABAergic synapse system, which may be involved in behavioral changes in this study.

S100B is the most extensively studied protein in the diagnosis and treatment of various conditions of CNS, and it can be used as a peripheral marker of brain damage, such as traumatic brain injury and stroke (42–45). 5-HT is a monoamine neurotransmitter which is found primarily in blood vessels, mammary glands and CNS (46, 47). Early evidence supported that 5-HT is considered as a key modulatory neurotransmitter involved in regulation of physiological and behavioral processes including anxiety-related behavior (46). In order to further investigate the potential mechanism of alteration of emotionality in mice induced by combined exposure to EMP and 4.9 GHz RF, we detected the concentration of S100B and 5-HT in serum. It was found that the level of S100B in serum was significantly up-regulated in 4.9 GHz RF single exposure and combined exposure groups, which suggested that brain injury occurred in the two groups. Moreover, the level of 5-HT in serum was significantly decreased after the combined exposure of EMP and 4.9 GHz RF. It was reported that 5-HT plays an important role in cognition, emotional behaviors and brain development (48, 49). Nevertheless, there are limited data about the effects of RF on serum level of 5-HT. Eris et al. (50) previously found that a single 45 min 900 MHz low level electromagnetic radiation by mobile phones caused an increase in blood 5-HT in rats. We believe the inconsistent results may be attributed to different exposure parameters and detecting method.

It was proved that hippocampus and amygdala play important role in regulating memory and emotionality in the brain (51–53). We then observed the histology of hippocampus and amygdala, our results showed that 4.9 GHz RF single and combined exposure caused morphological alteration in amygdala, instead of hippocampus, meanwhile, the number of Nissl body in amygdala significantly decreased, which suggested that the histological damage of amygdala might be involved in 4.9 GHz RF single exposure and combined exposure induced emotional behavioral change, which was consistent with Narayanan's study (54).

To explore the way of neuronal death in amygdala, we detected the level of cell apoptosis and autophagy. It was found that the apoptosis level did not change after single or combined exposure to EMP and 4.9 GHz RF in hippocampus and amygdala. Autophagy is a protective cellular process, especially happens after acute damage, and excessive autophagy can also cause cell death (55). LC3-II is a specific marker of autophagosome, and it retains steadily in autophagosome membrane until lysed (18). It was found that after single exposure to 4.9 GHz RF and combined exposure, the level of autophagy significantly increased in amygdala instead of hippocampus, which was in consistence with histological results. However, the researches relating to the effects of EMF on autophagy were rare and consensus had not been reached. It was previously reported that repetitive frequency 100 Hz, 50 kV/m EMP daily exposure could induce autophagy in the hippocampus of SD rats (17, 18). Up to date, there is no available information on accumulative effect of such combined exposure, further investigations are warranted.

It is noted that according to International Commission on Non-Ionizing Radiation Protection (ICNIRP), the exposure level of 4.9 GHz RF (50 W/m^2^) is also closely related to occupational situation, as it has exceeded the exposure limit of general public (56). Occupational-exposed situations are not deemed to cause greater hazard than general public, due to appropriate screening and training are provided for known risks. Therefore, future study about exposure level within general public is warranted. In conclusion, in the present study, we revealed the effects of combined exposure to 4.9 GHz RF from 5G communications and EMP on emotional behavior, and found that combined exposure could induce anxiety-like behavior in mice, which might be related to Glutamatergic and GABAergic synapse system of hippocampus and autophagy in amygdala.

## Data availability statement

The datasets presented in this study can be found in online repositories. The names of the repository/repositories and accession number(s) can be found below: http://www.proteomexchange.org/, PXD038101.

## Ethics statement

The animal study was reviewed and approved by Animal Welfare Committee of Air Force Medical University (IACUC-20210105).

## Author contributions

TQ and LL performed the exposure and behavioral tests. JL contributed to exposure devises set-up and parameters estimation. TQ, LL, LG, JD, and YX did the experiments. PL and YJ helped analyze the data. TQ and XW wrote the manuscript. GD contributed to the idea, design of the study, and revised the manuscript. All authors agree to be accountable for the content of the work.
